# Sex Ratio at Birth after Concurrent Events of Earthquakes and the COVID-19 Pandemic in Croatia

**DOI:** 10.3390/ijerph21050572

**Published:** 2024-04-29

**Authors:** Dario Pavić

**Affiliations:** Department of Sociology, Faculty of Croatian Studies, University of Zagreb, Borongajska Cesta 83d, 10000 Zagreb, Croatia; dpavic@fhs.hr

**Keywords:** sex ratio at birth, earthquake, COVID-19, Croatia, stress

## Abstract

The sex ratio at birth (ratio of males to females) has been known to be affected by exogenous shocks such as wars, pollution, natural catastrophes, economic crises, and others. Among these stressful events, both earthquakes and the COVID-19 pandemic have been reported to lower the sex ratio at birth. In this article, a rather unusual situation of two episodes of simultaneous events of COVID-19 lockdown and earthquakes approximately nine months apart (March and December of 2020) is investigated to assess whether they were associated with a bias in sex ratio at birth 3–5 months later (in utero loss) and 9 months later (loss at conception) in Croatia. The monthly time series of sex ratio at birth, total number of births, and total number of both male and female births from January 2010 to December 2021 were analyzed. Seasonally adjusted autoregressive moving-average models were used to estimate the functional form of the time series from January 2010 to February 2020. These results were used to predict the future values of the series until December 2021 and to compare them with the actual values. For all series used, there was no indication of deviation from the values predicted by the models, neither for 3–5 months nor for 9 months after the COVID-19 lockdown and earthquake events. The possible mechanisms of the absence of bias, such as the threshold of the stressful events and its localized reach, as well as the statistical methods employed, are discussed.

## 1. Introduction

The sex ratio at birth (SRB) is defined as the ratio of male live births and female live births over a period of time and is usually constant, approximately 1.06–1.07, indicating a slight excess of male births. Changes in the SRB have been observed in a multitude of stressful circumstances, including environmental pollution and certain occupations [1], natural catastrophes such as earthquakes [2], terrorist attacks and riots [3,4], war [5,6,7], socio-economic hardship [8], and many others. The literature on the departures from the usual value of SRB is vast and spans several decades, with results often being conflicting and underlying physiological mechanisms not completely elucidated. Here, only a broad and undetailed picture of the association of stressful events and SRB will be presented, with an emphasis on the health-related issues concerning the biased SRB.

Ever since the seminal research by Trivers and Willard [9], a bias in SRB has been associated with the condition of the mother (i.e., health status), with mothers in poor conditions having maximized reproductive success when siring female offspring, while for those mothers in better conditions, the advantage is in producing male offspring. The authors posit that this ability is favored by natural selection. Environmental stress affects the maternal physiology, enabling the conception and/or the survival of a fetus of a certain sex to be favored. It has been shown that stress disrupts the normal functioning of the HPG (hypothalamic–pituitary–gonadal) axis in females [10], which in turn affects the SRB. Similarly, the differential survival in utero could be mediated by low levels of progesterone in women exposed to acute stress [11]. Maternal inflammation has also been associated with fetal loss, miscarriage, and other pathological conditions in pregnancy. The research reviewed by Abdoli [12] suggested that both psychosocial and other types of stress increase inflammation markers in mothers and lead to more adverse effects for male offspring.

The adverse health conditions are not only related to biased SRB due to the mothers’ physiology, but there is evidence that fathers, especially those exposed to environmental toxins, also produce offspring with biased SRB [1]. The same is true for fathers diagnosed with testicular cancer and multiple sclerosis [13]. Regardless of the sex of the parent, those who have contracted hepatitis B produce more sons, and women positive for cytomegalovirus bear more daughters [13]. Biased SRB has been recorded in a multitude of pathological obstetric conditions [13] and in diabetic mothers [14]. 

Two main hypotheses aim to explain the bias in sex ratio at birth under stressful conditions. The “selection in utero” hypothesis posits that mothers exposed to stressful events tend to spontaneously abort frail male fetuses to maximize their fitness and avoid maternal investment that would be needed to sustain these frail male fetuses up to their reproductive age [15,16]. This results in decreased SRB, and the critical period for this selection is the second trimester of pregnancy; thus, the decreased SRB is exhibited 3–6 months after the stressful event. On the other hand, the “conception hypothesis” states that the bias in SRB happens at the time of conception, where stress modulates the levels of sex hormones, favoring one sex over the other. The SRB is a function of the coital rate, with a less frequent coital rate resulting in female-biased SRB, since a more frequent coital rate favors conception earlier in the fertile period, which in turn favors male conceptions [13,17]. The result of this hypothesis is a biased SRB 8–10 months after the stressful event.

The aim of the present study is to determine whether there was a change in SRB after two concurrent events of COVID-19 pandemic lockdowns and earthquakes in Croatia, and so the focus of the literature review will be on the effects of COVID-19 and earthquakes on SRB. The COVID-19 pandemic is considered a stressor that could bias the SRB [12], and recent reports found a decline in SRB three months after the onset of the COVID-19 pandemic in South Africa [18] and England and Wales, with an increase in SRB nine months after the onset of COVID-19 in the latter case [19]. In Iran, the SRB in the first nine months after the beginning of COVID-19 pandemic was not different than that in the pre-pandemic period, but in the period 10–13 months after the onset of the pandemic, the SRB was significantly higher compared to the pre-pandemic (control) period [20]. In most of the studies on the effect of earthquakes on SRB, a decline has been observed, except for the Wenchuan earthquake in China [21]; however, the intervals at which SRB declined varied significantly. A decline nine months after the earthquake was observed after the Kobe earthquake in Japan [22] and in Italy [23], while three-month declines occurred in Chile [24] and Tohoku, Japan [25]. There are also reports of declines in SRB 4, 8, 7, 10, and 11 months after earthquakes (for a comprehensive review, see [2]). 

In March 2020, measures against the spreading of COVID-19 were instituted by the Government of the Republic of Croatia. Social gatherings, commerce, service, and sports events were restricted and leaving one’s usual place of residence was prohibited, being allowed only in special circumstances with a permit. This restriction of movement was abolished in May 2020. With the onset of the second wave of COVID-19 infections, some of these measures were re-instituted, most notably the prohibition of leaving one’s usual place of residence (all official statements about the COVID-19 pandemic in Croatia are available at the official website [26]). 

Almost immediately after the anti-COVID-19 measures were instituted, an earthquake occurred on 22 March 2020 at 6:24 a.m., with a magnitude of 5.5 on the Richter magnitude scale, with the epicenter 5 km east of Gornja Bistra, near the Capital of Zagreb [27]. One person was killed and twenty-seven persons injured, while more than 26,000 buildings were damaged and 1900 of them deemed unusable, mostly in the historic center of Zagreb. By 14 April 2020, almost one thousand aftershocks had been recorded [28]. On 20 December 2020, at 12:20 p.m., during the second wave of measures against the COVID-19 pandemic, another earthquake occurred 3 km outside of the town of Petrinja, approximately 50 km southeast of Zagreb, with a magnitude of 6.2 on Richter magnitude scale [29]. Seven people were killed and twenty-six injured, along with significant damage to nearby towns and villages and numerous aftershocks. The total estimated damage was EUR 5–5.5 billion [30]. 

The unlikely concurrence of the earthquake and the COVID-19 pandemic wave twice during the same year (March and December 2020) represents a unique opportunity to test both the “selection in utero” and “conception” hypotheses of the decline in SRB. Under the “selection in utero” hypothesis, SRB should show a decline 3–5 months after each of the concurrent events, while a similar decline should occur after nine months under the “conception” hypothesis.

## 2. Materials and Methods

To assess the possible impact of two COVID-19 pandemic episodes simultaneous with two earthquakes in 2020 in Croatia, the analysis of a time-series of the sex ratio at birth was employed, similarly some recent studies [18,19,31]. The monthly number of male, female, and total births from January 2010 to December 2021 was obtained from the publicly available database of the Croatian Bureau of Statistics [32]. The sex ratio at birth was calculated as the number of males per one female. The pre-pandemic data on the sex ratio at birth from January 2010 until February 2020 were used to predict the series from March 2020 onwards by finding the best-fitting autoregressive moving average (ARIMA) model (the lowest AIC statistic), considering the seasonality of the monthly SRB data. The ARIMA method allows the times series to be modeled in terms of the autoregressive (AR) and moving average (MA) components. The AR component models the relationship between an observation and the number of lagged observations (previous time steps). It assumes that the current value of the series is a linear combination of its past values. The integrated component (I) refers to differencing the raw observations (subtracting an observation from the observation at the previous time step) to make the time series stationary. Finally, the moving average component models the relationship between an observation and a residual error from a moving average model applied to lagged observations. It is usually referred to with three parameters (*p*, *d*, *q*) in parentheses, where *p* stands for the number of lagged observations included in the AR model, *d* stands for the degree of differencing applied to make the series stationary, and *q* represents the size of the moving average window. In the presence of seasonality in the time series, an additional three parameters are modeled similarly to those mentioned, but only for the seasonal component of the series (*P*, *D*, *Q*). 

Based on the best-fitting model, a 95% prediction interval was constructed for the data from March 2020 to December 2021. The values outside this interval 3–5 months or 9 months after each event would have indicated an effect of this event on the SRB. To address the fact that a decline in the number of male births due to the effect of the stressful event could be offset by a decline in female births due to preterm delivery [24], the same time-series modeling was used on the male and female birth series, respectively, to assess the possible concurrent decline in the number of female and male births. Since microdata on every birth were not available, the exact approach of [24] could not be replicated. To test whether there was a culling of males in utero 3–5 months after the stressful events, the periods of June to August 2020 and March to May 2021 were chosen, respectively. For the test of differential conception at birth, periods of nine months after the stressful events were chosen (December 2020 and September 2021). The analyses were performed in the R programming language (version 4.3.0), with the “forecast” package.

## 3. Results

**Figure 1:**
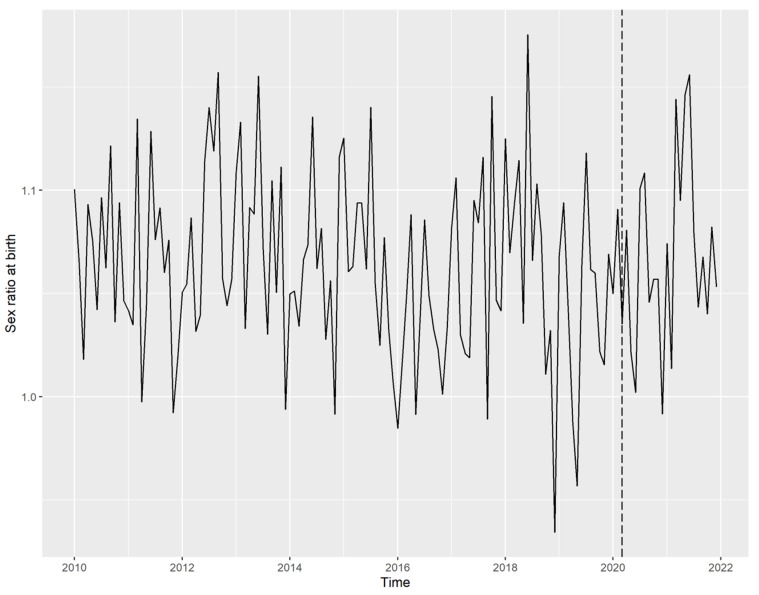
Time series of sex ratio at birth in Croatia from January 2010 to December 2021. Note: Dashed vertical line represents March 2020—the onset of COVID-19 pandemic and the month of the first earthquake.

In the analyzed period (January 2010 to December 2021), a total number of 462,863 live births were recorded, of which 238,601 were males and 224,262 were females, yielding a total SRB of 1.064. The complete monthly series of SRB is presented in Figure 1. 

**Figure 2:**
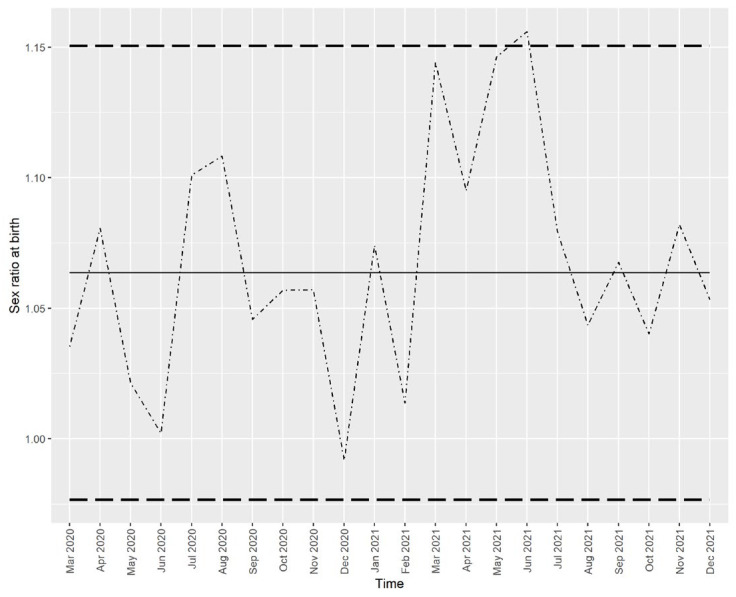
The actual and predicted values of sex ratio at birth in Croatia from March 2020 to December 2021. Note: Solid line—prediction from the time series model, dotted/dashed line—the actual values, dashed lines—the limits of the 95% prediction interval.

The best-fitting model for the series from January 2010 to February 2020 is the ARIMA (0, 0, 0) model, i.e., the pure white noise process without any systematic components. Based on this model, a prediction of the next 22 monthly values and 95% prediction intervals is shown in Figure 2. 

Since the time-series model is a purely random process, the predicted values are just the mean of the series (solid line), and the 95% prediction intervals are constant over time. It is evident that, for the critical periods for both the “culling” (3–5 months after March 2020) and “conception” (nine months after March 2020) hypotheses, the SRB remained within the prediction intervals, i.e., there was no significant deviation of the SRB from the predicted values. In December 2020, nine months after the stressful event, a decline in SRB is visible, although it is not statistically significant. The only value outside of the prediction interval is the one from June 2021. This finding will be discussed later.

**Figure 3:**
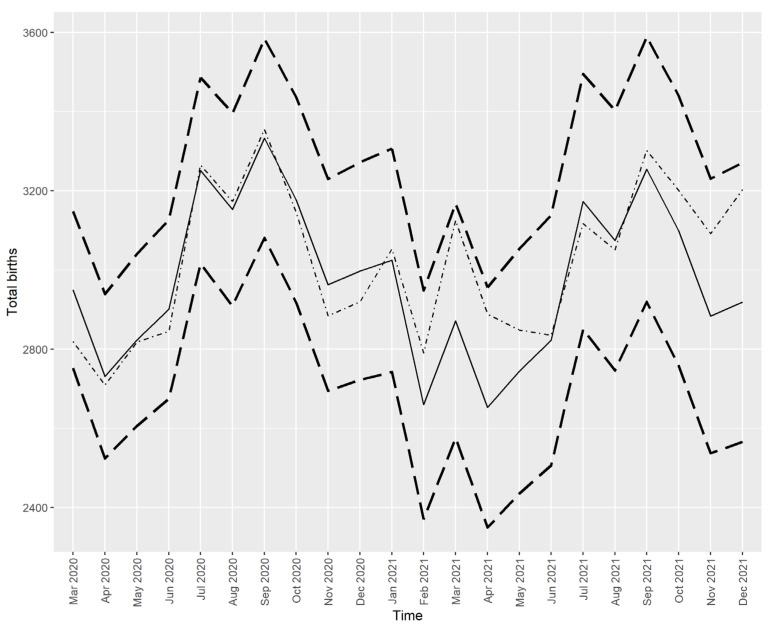
The actual and predicted values of total live births in Croatia from March 2020 to December 2021. Note: Solid line—prediction from the time series model, dotted/dashed line—the actual values, dashed lines—the limits of the 95% prediction interval.

The best-fitting model for the total number of births was the ARIMA (1, 0, 1) (2, 1, 0) seasonal model with drift, which produced the predicted values shown in Figure 3. 

The total number of births (dotted/dashed line) closely follows the predicted values (solid line), except for number of births in March 2021, where the actual value is somewhat higher than the predicted one; however, the actual value does not fall out of the prediction interval.

**Figure 4:**
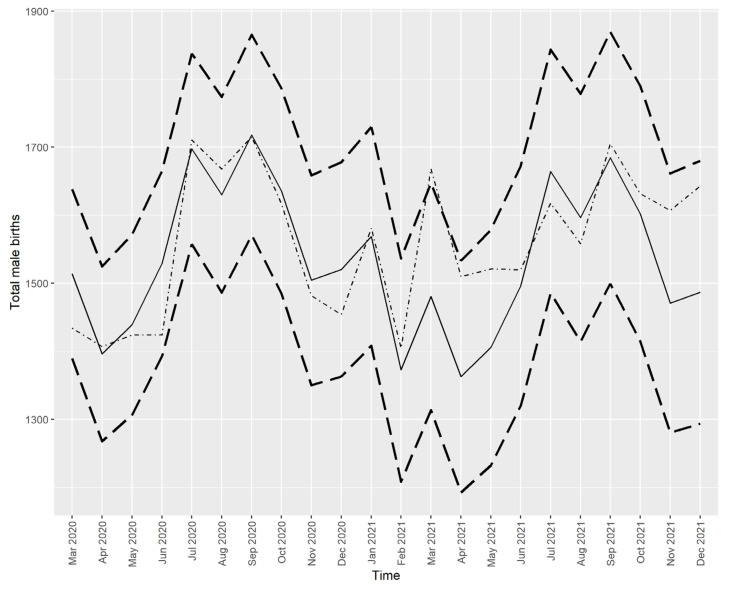
The actual and predicted values of male live births in Croatia from March 2020 to December 2021. Note: Solid line—prediction from the time series model, dotted/dashed line—the actual values, dashed lines—the limits of the 95% prediction interval.

**Figure 5:**
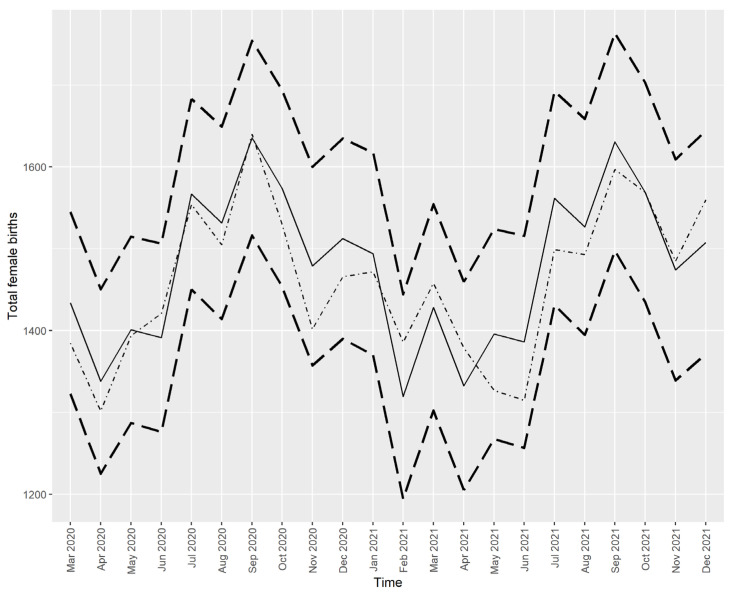
The actual and predicted values of female live births in Croatia from March 2020 to December 2021. Note: Solid line—prediction from the time series model, dotted/dashed line—the actual values, dashed lines—the limits of the 95% prediction interval.

The best fitting models for the series of male births and female births are ARIMA (3, 0, 0) (2, 1, 0) with drift and ARIMA (0, 0, 0) (1, 1, 0) with drift, respectively. Figure 4 and Figure 5 show the predicted values for male births and female births, respectively.

Figure 4 and Figure 5 show that all the actual values (dotted/dashed lines) were within the 95% prediction interval and close to the predicted values (solid lines), except for the number of male births in March 2021, which was higher than predicted. However, this higher-than-expected number of male births did not drive the SRB value out of the predicted interval, as seen in Figure 2.

## 4. Discussion

The results of this study suggest that there was no statistically significant bias in the sex ratio at birth after the stressful events of the COVID-19 pandemic and earthquakes in Croatia. The one SRB value outside of the prediction interval (June 2021, higher SRB) cannot be taken as a basis for rejecting the null hypothesis. This value is out of the critical period defined in the hypotheses, and its direction is opposite to the expected one (i.e., a decline). The 95% prediction interval suggests that, on average, 1 in 20 values would lie outside of this interval even without the true effect. Since this study predicted 22 values, it is most likely that this outlying value was due to chance. This is most probably true for the one outlying value in the series of predicted male births. There is also no indication that the unbiased values of SRB were the result of a concurrent decline in both male and female births, as suggested by [24]. The series of both male births and female births was close to the predicted values, with no observable concurrent declines.

The body of evidence suggests that the sex ratio at birth in Croatia is quite constant and unresponsive to shocks. Even though the war is often cited as a possible cause of the bias in the SRB [5,33], no statistically significant change in the SRB due to war was found during the 1991–1995 war in Croatia [6]. Also, no secular trend in SRB was observed in Croatia from 1946 to 2011, and the annual values of this series are also the product of a white noise process [34], even though changes in the SRB have been documented for a significant number of countries in a similar time period [35]. 

It is difficult to discuss the potential causes of the absence of an effect, but the presence of psychological stress was documented during the COVID-19 pandemic and after the first earthquake in Croatia, with almost 16% of respondents reporting severe to extreme depression, almost 11% severe to extreme anxiety, and 26.2% severe to extreme stress [36]. Yet, it has been posited in other studies on the effect of stressful events on the SRB that a certain threshold of stress must be surpassed for the effect to be detectable [37,38]. This could explain the lack of bias in the SRB during the studied period in Croatia, but it remains unclear how the measure of this threshold could be established and why it has supposedly been surpassed in some other countries, but not in Croatia. Partial additional support for the lack of an effect of severe stress on birth outcomes in Croatia comes from the data from the largest maternity clinic in the capital, Zagreb. Even though the still-birth rate doubled from 9 per 1000 births to 18 per 1000 births, and the number of extremely premature deliveries (<28 gestational weeks) increased from 28 to 43, the other pregnancy outcomes (gestational age, preterm delivery over 28 weeks, birth weight, etc.) did not change significantly from 2019 to 2020 [39]. It has been reported that the elevated SRB in Iran could be a result of increased sexual activity seen as a coping mechanism against the elevated levels of anxiety during the pandemic period [20]. Whether this mechanism could serve as an explanation of why the SRB in Croatia did not decline will remain speculative without further knowledge of the sexual behavior of the Croatian population before and during the studied period.

It must be noted that the present study is not the only one that has failed to detect an effect of the stressful event on SRB. In the most recent systematic review of the effects of catastrophic events on the sex ratio at birth, out of the 25 eligible studies, sixteen of them found a decline in the SRB, three detected a rise in SRB, four did not detect any changes, and two studies detected contradictory results [40]. Grech [41] reviewed (and co-authored) several studies where no effect on SRB was found, possibly due to a small cohort size or the ability of the population to adapt to stressful events. One could argue that not all of the Croatian population was equally affected by the earthquake and the COVID-19 pandemic, and that a subpopulation of the most-affected individuals could have been affected, as was the case of the 2011 earthquake in Japan [42]. However, the stricken area would be difficult to define geographically, and sub-national statistics on monthly births are not publicly available. Also, when comparing this study with the other ones, especially those on the effect of earthquakes on SRB, special attention should be directed toward the statistical methods used to assess the effect. Some of the studies [21,22,43,44] used a simple cohort comparison approach wherein the exposed group was statistically compared with an unexposed one, usually in the past. This approach has been criticized because it does not take into account temporal trends, seasonal variations, or short-term fluctuations [40,45]. The time series approach used in this study can solve these issues, and although it is not without possible flaws [45], it is still preferable to a simple cohort analysis. A particularly informative example of using proper statistical techniques to assess the possible biases in SRB is the study by Schnettler and Klüsener [46], which revisits the analysis of the effect of economic stress on SRB after the reunification of Germany. While another study found a decreasing SRB in 1991 after the reunification [47], the study by Schnettler and Klüsener, using the time-series approach and individual-level data analysis, demonstrated that the supposed bias in SRB was the product of random variation, rather than the effect of economic stress. This cautionary example stresses an additional shortcoming of most of the studies on the effect of stressful events on the SRB, i.e., there is a multitude of possible confounding factors that occurred at the same time as the stressful event and that are usually not empirically controlled for [46]. This is also a shortcoming of the present study, since, due to the unavailability of individual-level data, it is not possible to account for the possible confounding factors, such as economic situation, coital rate, age, exposure to toxins, etc. 

Another important factor contributing to the dynamics of SRB is the timing and the duration of the stressful event, i.e., the short-term and long-term effects of stress. In a model proposed by Grant and collaborators [48], higher levels of follicular testosterone and glucose were found to be associated with stress and also with the probability of conceiving male offspring. However, prolonged stress tends to negatively affect male offspring, leading to culling of male embryos in utero. Based on this model, it is predicted that, if stress occurs at the time of conception, more males will be produced, but if the stress persists, more male attrition will happen during pregnancy, and the SRB will ultimately remain normal. Likewise, if stress occurs only around conception, the SRB will be male-biased, and if it occurs during pregnancy, it will be female-biased [46]. It can be postulated that the earthquake-induced stress was short-term, but the COVID-19 stress lasted for a significant number of months. However, the combination of short- and long-term stress that produces a normal SRB usually manifests itself in annual data, not in monthly data, so in the present analysis, this effect is not visible. Rather, keeping in mind the constraints mentioned previously, it appears that the dynamics of SRB in the studied period in Croatia are most likely a product of random variation. Therefore, due to different population characteristics, stress intensity and timing, cohort sizes, and methods of analysis, a clearer picture of the effects of the stressful event on the SRB remains elusive. As suggested by Song [45], future research on the effects of stress on the SRB should rely on natural experiments with the use of “difference-in-difference” or time-series analysis, with more attention directed toward the identification of the underlying mechanism through which stress influences SRB. 

## 5. Conclusions

While some of the studies did find an effect of COVID-19 and earthquakes on the SRB, Croatia’s SRB remained unbiased in the case of two concurrent episodes of both COVID-19 and earthquakes. Also, there was no change in the series of total live births, nor in the total male and female births during the studied period. The literature on biased SRB after stressful events is abundant, yet the results are mixed, with a single comprehensive explanation still lacking. Additionally, some mentioned studies, similarly to the present one, failed to detect any significant effect of stress on SRB. This raises a question regarding the nature of the effect of stress on SRB itself, mainly through the threshold of stress needed to produce the effect and its geographical reach, as well as the timing of the stressful event. The plethora of faux findings and the possible publication bias, along with several well-designed studies that challenge the evidence of biased SRB during stressful events, serve as initial evidence that the claimed effects might be artifacts, or, at least, not nearly as strong as is claimed. Special attention should be directed towards the statistical methods used to assess the effect of stress on SRB, with some good practices already being established.

## Data Availability

The data are publicly available through the web page of Croatian Bureau of Census, http://web.dzs.hr/ (accessed on 15 June 2023).
